# The effects of acute incremental hypocapnia on the magnitude of neurovascular coupling in healthy participants

**DOI:** 10.14814/phy2.14952

**Published:** 2021-08-04

**Authors:** Taylor J. Bader, Jack K. Leacy, Joanna R. G. Keough, Anna‐Maria Ciorogariu‐Ivan, Joshua R. Donald, Anthony L. Marullo, Ken D. O’Halloran, Nicholas G. Jendzjowsky, Richard J. A. Wilson, Trevor A. Day

**Affiliations:** ^1^ Department of Biology Faculty of Science and Technology Mount Royal University Calgary AB Canada; ^2^ Department of Physiology School of Medicine College of Medicine and Health University College Cork Cork Ireland; ^3^ Division of Respiratory and Critical Care Physiology and Medicine The Lundquist Institute for Biomedical Innovation at Harbor‐UCLA Medical Center Torrance CA USA; ^4^ Department of Physiology and Pharmacology Hotchkiss Brain Institute Cumming School of Medicine University of Calgary Calgary AB Canada

**Keywords:** hypocapnia, neurovascular coupling, respiratory alkalosis

## Abstract

The high metabolic demand of cerebral tissue requires that local perfusion is tightly coupled with local metabolic rate (neurovascular coupling; NVC). During chronic altitude exposure, where individuals are exposed to the antagonistic cerebrovascular effects of hypoxia and hypocapnia, pH is maintained through renal compensation and NVC remains stable. However, the potential independent effect of acute hypocapnia and respiratory alkalosis on NVC remains to be determined. We hypothesized that acute steady‐state hypocapnia via voluntary hyperventilation would attenuate the magnitude of NVC. We recruited 17 healthy participants and insonated the posterior cerebral artery (PCA) with transcranial Doppler ultrasound. NVC was elicited using a standardized strobe light stimulus (6 Hz; 5 × 30 s on/off) where absolute delta responses from baseline (BL) in peak, mean, and total area under the curve (tAUC) were quantified. From a BL end‐tidal (P_ET_)CO_2_ level of 36.7 ± 3.2 Torr, participants were coached to hyperventilate to reach steady‐state hypocapnic steps of Δ‐5 Torr (31.6 ± 3.9) and Δ‐10 Torr (26.0 ± 4.0; *p* < 0.001), which were maintained during the presentation of the visual stimuli. We observed a small but significant reduction in NVC peak (ΔPCAv) from BL during controlled hypocapnia at both Δ‐5 (−1.58 cm/s) and Δ‐10 (−1.37 cm/s), but no significant decrease in mean or tAUC NVC response was observed. These data demonstrate that acute respiratory alkalosis attenuates peak NVC magnitude at Δ‐5 and Δ‐10 Torr P_ET_CO_2_, equally. Although peak NVC magnitude was mildly attenuated, our data illustrate that mean and tAUC NVC are remarkably stable during acute respiratory alkalosis, suggesting multiple mechanisms underlying NVC.

## Graphical abstract

We assessed the NVC response to a visual stimulus, under conditions of normocapnia and two steps of hypocapnia via voluntary hyperventilation.

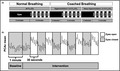

## 1 INTRODUCTION

The human brain constitutes merely 2% of total body weight, yet is responsible for approximately 20% of total oxygen consumption at rest (Mergenthaler et al., 2013). To ensure that cerebral metabolic demand is met, the brain requires sophisticated and well‐regulated global and regional vascular perfusion. The spatial and temporal coordination between regional cerebral blood flow and metabolic demand is termed neurovascular coupling (NVC). NVC is mediated at the level of the neurovascular unit, and involves the interplay between astrocytes, neurons, vascular smooth muscle, and endothelial cells, as well as pericytes at the capillary level (Phillips et al., 2016; Willie et al., 2014), matching regional CBF to regional increases in neuronal activity in order to reliably meet metabolic demands (Fernández‐Klett et al., 2010; Phillips et al., 2016; Rosengarten et al., 2003). Astrocytes use their podocytes to modulate blood flow to metabolically active areas by bridging synaptic activity with metabolic activity (Blanco et al., 2008). The release of nitric oxide (NO) from both endothelial cells and astrocytes causes vasodilation in downstream vasculature, resulting in an increase in regional flow (Crecelius et al., 2011; Fathi et al., 2011; Hoiland et al., 2020). Controlling the spatial distribution of NVC allows greater precision in matching fluctuating local metabolic rates to vascular perfusion (Fernández‐Klett et al., 2010). Experimentally, NVC is commonly monitored non‐invasively via ultrasound of the posterior cerebral artery (PCA) due to its relatively small territory of perfusion involved in visual processing tasks (i.e., occipital lobes; Sturzenegger et al., 1996).

Furthermore, cerebrovascular reactivity (CVR) contributes to the regulation of cerebral blood flow, through the modulation of vessel tone, in response to perturbations in blood gases (e.g., CO_2_ and O_2_; Kim et al., 2015; Phillips et al., 2016; Willie et al., 2014). During blood gas perturbations, CVR is vital for maintaining cerebral homeostasis through adequate perfusion. Cerebrovascular tone is particularly sensitive to changes in arterial CO_2_ (P_a_CO_2_). A decrease in PaCO_2_, termed hypocapnia, induces the vasoconstriction of the cerebrovasculature. Furthermore, hypocapnia, particularly in the acute setting, can induce a profound acid–base disturbance resulting in progressive increases in arterial pH (pHa), with resultant alkalosis (Krapf et al., 1991; Norcliffe et al., 2005; Poulin et al., 2002). In contrast, reductions in O_2_ have a vasodilatory effect. Instances of acute and chronic hypoxia cause both vasodilation in cerebral blood vessels and an increase in ventilation, the latter termed the hypoxic ventilatory response (HVR; Teppema & Dahan, 2010). The HVR raises the partial pressure of arterial O_2_ (PaO_2_), while decreasing the partial pressure of arterial CO_2_ (PaCO_2_), and thus the pressure of end‐tidal PCO_2_ (P_ET_CO_2_).

The relationship between acute hypocapnia and/or respiratory alkalosis and NVC remains unclear. Prior research has investigated the effects of chronic and incremental hypocapnia on NVC during high altitude (HA) ascent with ventilatory acclimatization (Caldwell et al., 2017; Leacy et al., 2018). However, confounding variables of concomitant hypoxia and renal compensation of pH through bicarbonate (HCO_3_^−^) elimination exists in these studies (Krapf et al., 1991; Zouboules et al., 2018). Both studies found that NVC was stable during steady‐state or incremental ascent (Caldwell et al., 2017; Leacy et al., 2018), potentially explained by conflicting cerebrovascular stimuli. In contrast with the results found at altitude, in a laboratory setting, Szabo et al. (2011) found that peak NVC magnitude decreased in acute hypocapnic conditions. However, this study only utilized a single hypocapnic step through coached hyperventilation, as opposed to targeting relative CO_2_ values. In addition, Szabo et al. (2011) assessed the magnitude of the NVC response using a single metric: %‐change in peak responses. What remains to be tested are the independent effects of controlled and graded hypocapnic steps without the confounding effects of superimposed and conflicting hypoxia and renal compensation. The aim of this study was to determine whether NVC response magnitude is affected during acute, incremental hypocapnia, and respiratory alkalosis in a laboratory setting, when compared with normocapnia. We hypothesized that there will be step‐wise decreases in NVC magnitude during each step of acute hypocapnia, specifically Δ‐5 and Δ‐10 Torr P_ET_CO_2_ from eupnoeic values.

## 2 METHODS

### 2.1 Ethics approval and participant recruitment

This study abided by the Canadian Government Tri‐Council Policy on research with human participants, consistent with the Declaration of Helsinki, except for registration in a database. Ethical approval was received from the Mount Royal University Human Research Ethics Board (Protocol 101879) and the University of Calgary Conjoint Health Research Ethics Board (Protocol REB18‐0374) in advance. Participants were informed of the experimental procedures and provided written and verbal consent prior to data collection. Participants completed a detailed pre‐medical questionnaire, which was used to assess for any contraindications to study participation, prior to data collection. Participants were otherwise healthy with no previous or current medical history of cardiovascular, respiratory, neurological, and/or metabolic disease. In addition, participants with a body mass index of >30 kg/m^2^ or a history of epilepsy and/or seizures were excluded. Participants were asked to abstain from consuming caffeine for at least 12 h prior to data collection.

### 2.2 Instrumentation

Participants remained in the seated position throughout data collection. Participants were instrumented with a Finometer (Finometer Pro, Finapres Medical Systems; calibrated using the return to flow function for every participant), lead II electrocardiogram (ECG; ADI bioamp ML132), nose clip, mouthpiece, spirometer (800 L flow head and spirometer amplifier, 3813 series, Hans Rudolph, Shawnee, HS; and ADI ML141; calibrated with a 3‐L syringe), dual O_2_ and CO_2_ gas analyzer (ADI ML206; calibrated daily), and transcranial Doppler ultrasound (TCD; PMD150B, Spencer Technologies). Mean arterial pressure (MAP) and heart rate (HR) were derived from the raw Finometer waveform and ECG signal, respectively. Specifically, MAP was calculated as the mean of the raw Finometer channel, and HR was calculated as 60/period from the ECG signal. The spirometer measured respiratory flow, with the resulting inspired minute ventilation (V̇_I_) calculated as the product of respiratory rate and inspired volume (calculated as the integral of the inspired flow). The gas analyzer measured CO_2_ and O_2_ in %, and P_ET_CO_2_ and P_ET_O_2_ were calculated and corrected for body temperature, atmospheric pressure, and saturated with water vapor (BTPS; ~669 mmHg P_ATM_ in Calgary, 1130 m).

Using standardized TCD insonation procedures (Willie et al., 2011), we located and measured beat‐by‐beat cerebral blood velocity (CBV) through the PCA. The PCA was insonated on either the right or left side of the cranium, dependent upon the quality of the transtemporal acoustic window, allowing for the accurate measurement of beat‐by‐beat changes in CBV. Aside from accepted insonation criteria (e.g., probe placement, depth, mean velocity), a series of standardized techniques and functional tests (e.g., carotid compression and visual stimulation (VS)) were also used to confirm PCA insonation (Willie et al., 2011). Using TCD, NVC magnitude through the PCA can be measured as the change in CBV in response to VS, owing to the perfusion territory of the PCA within the occipital cortex, where visual processing occurs (Phillips et al., 2016; Willie et al., 2011).

### 2.3 Protocol

**FIGURE 1:**
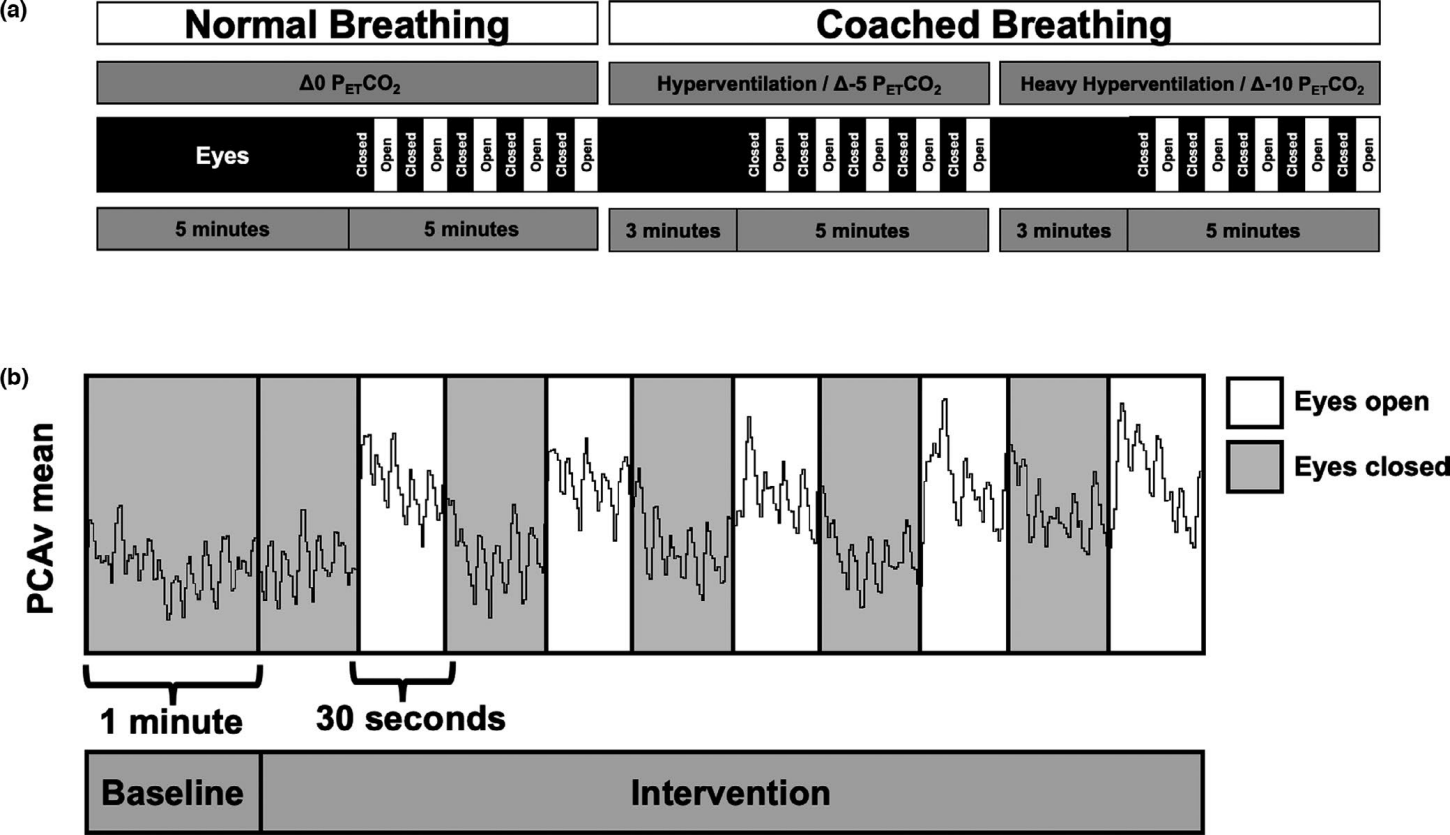
Protocol schematic and sample tracing. (a) Protocol schematic. There was a total of five 30‐s strobe light trials at resting P_ET_CO_2_ and both hypocapnic conditions. Breathing was coached during each intervention to reach targeted P_ET_CO_2_ (‐5 and ‐10 Torr). The order of hyperventilation and heavy hyperventilation trials was randomized between participants. (b) Raw tracing during one set of five light stimuli trials in a single participant. Visual stimulation was elicited with a 6 Hz strobe light, six inches from their eyes. Grey shaded regions denote periods of baseline and rest between VS trials with eyes closed

Following recruitment and pre‐screening, participants reported to the lab for a ~ 2‐h protocol between 10:00 am and 8:00 pm. Participants sat in a dimly lit, quiet room for 5‐min with their eyes closed to reach steady‐state before 1‐min of relaxed Δ 0 Torr P_ET_CO_2_ baseline (BL) data were recorded. Following Δ 0 Torr P_ET_CO_2_ BL, a standardized NVC assessment was carried out. NVC assessment involved a series of five intermittent 30‐s on/off visual stimulus (VS) trials using a flashing strobe light (6 Hz; Apple iPhone), held approximately 15 cm directly in front of the participant's eyes. Two step‐wise hypocapnic interventions, Δ‐5 and Δ‐10 Torr P_ET_CO_2_, were then performed in a randomized order. Individuals hyperventilated until their P_ET_CO_2_ was at the targeted value from their respective normocapnic P_ET_CO_2_ (Δ 0 Torr P_ET_CO_2_). Between 0, Δ‐5, and Δ‐10 Torr P_ET_CO_2_, participants were coached to increase or decrease ventilation until they reached the desired P_ET_CO_2_, which was maintained throughout the protocol with verbal cues as needed to maintain their P_ET_CO_2_ at ±1 Torr for the duration of the NVC trial. An initial 1‐min BL was collected for each participant at each condition. The mean of this BL was used to compare with the average peak and mean of the five 30‐s intervals of eyes open during the VS intervention. The Henderson–Hasselbalch equation was utilized to estimate pHa in each participant from P_ET_CO_2_ and normative [bicarbonate] values in Calgary (Krapf et al., 1991; Zouboules et al., 2018). Participants kept their eyes closed throughout the protocol unless told to open them during the VS for NVC assessment. The schematic of the protocol is illustrated in Figure 1a, with a representative NVC tracing in Figure 1b.

### 2.4 Data analysis

All data were collected using a 16‐channel PowerLab system with a sampling frequency of 200 Hz (PowerLab/16SP ML880; AD Instruments; ADI) and analyzed offline using commercially available software (LabChart Pro software v8.0), later analyzed offline using Microsoft Excel and Sigma Plot (SigmaPlot v14, Systat Software, Inc.).

#### 2.4.1 Baseline data

One‐factor repeated measures ANOVA (1F RM ANOVAs) was used to assess for any significant differences in BL variables for each gas trial (Table 1). When significant *F*‐ratios were detected, Student–Newman–Keuls post hoc tests were used for pair‐wise comparisons.

**TABLE 1:** Cardiorespiratory and cerebrovascular parameters of all participants during baseline across each condition. Heart rate (HR) and mean arterial pressure (MAP) were not significantly different between each condition. Inspired minute ventilation (V̇_I_) increased with each intervention, with concomitant increases in P_ET_O_2_. In addition, partial pressure of end‐tidal CO_2_ (P_ET_CO_2_), posterior cerebral artery velocity (PCAv), PCA conductance (PCAcvc), significantly decreased with each intervention compared to Δ 0 Torr condition

Variable	Δ 0 Torr P_ET_CO_2_	Δ‐5 Torr P_ET_CO_2_	Δ‐10 Torr P_ET_CO_2_
HR (min^−1^)	76.9 ± 6.1	76.4 ± 12.1	81.5 ± 11.9
MAP (mmHg)	102.9 ± 11.8	102.4 ± 12.6	103.3 ± 9.3
PCAv (cm/s)	39.3 ± 8.6	36.2 ± 7.3*	30.7 ± 6.4* ^,^ ^‡^
PCAcvc (cm/s/mmHg)	0.44 ± 0.09	0.40 ± 0.09*	0.35 ± 0.07* ^,^ ^‡^
V̇_I_ (L/min)	12.9 ± 2.8	15.7 ± 3.3*	20.6 ± 5.7* ^,^ ^‡^
P_ET_O_2_ (Torr)	84.2 ± 7.0	87.9 ± 7.3*	102.9 ± 5.4* ^,^ ^‡^
P_ET_CO_2_ (Torr)	36.7 ± 3.2	31.6 ± 3.9*	26.0 ± 4.0* ^,^ ^‡^
Estimated pHa	7.43 ± 0.04	7.46 ± 0.06*	7.58 ± 0.08* ^,^ ^‡^

* Indicates a significant difference from Δ 0.

‡ Indicates a significant difference between Δ‐5 and Δ‐10 Torr P_ET_CO_2_ interventions. Values are presented as mean ± SD.

#### 2.4.2 NVC responses

From the raw and derived mean PCAv channel, NVC magnitude was analyzed using four distinct metrics: (a) peak, the highest PCAv during each VS trial, wherever it occurred, compared with BL (i.e., ΔPCAv; averaged for all five trials, within‐individual), (b) time‐to‐peak, from the onset of VS to the visually identified peak, (c) mean, the average PCAv across the entire VS trial, compared with BL (i.e., ΔPCAv; averaged for all five trials, within‐individual), and (d) total area under the curve (tAUC), the combined tAUC of the 30‐s VS, compared with a 30‐s pre‐VS BL (averaged for all five trials, within‐individual). Paired *t* tests were utilized to assess NVC magnitude by comparing BL and VS in each NVC metric for each gas trial to confirm the presence of an NVC response.

#### 2.4.3 Comparison of NVC responses

Last, 1F RM ANOVAs were utilized to assess ΔPCAv magnitude between Δ 0, Δ‐5, and Δ‐10 Torr P_ET_CO_2_ for each NVC metric (i.e., peak, time‐to‐peak, mean, and tAUC).

In all cases, significant differences were assumed at *p* < 0.05. All tests fulfilled the parametric assumptions of normal distribution and equal variance, with the exception of HR and P_ET_CO_2_ during BL (Table 1), where paired t‐tests were performed on ranks (i.e., non‐parametric).

## 3 RESULTS

### 3.1 Participants

A total of 22 participants were recruited for the protocol. Five participant data files were either incomplete or were excluded from analysis due to some combination of: (a) Δ PCAv falling out of the 1.5× interquartile range (i.e., statistical outlier), (b) a non‐responsive PCA in normoxia, (c) withdrawal from discomfort, feeling ill from hyperventilation and/or headache from the TCD headpiece, (d) inability to target P_ET_CO_2_ with coached hyperventilation, and/or (e) poor signal quality in hypocapnia. A total of 17 participants (5 males and 12 females) were included for final data analysis. These participants had a mean age of 23.2 ± 3.8 and a mean body mass index of 23.2 ± 2.9 kg/m^2^.

BL cardiorespiratory values are reported in (Table 1). At Δ 0, Torr P_ET_CO_2_ participants had an average HR of 76.9 ± 6.1 BPM, which was not significantly different than 76.4 ± 12.1 and 81.5 ± 11.9 during Δ‐5 and Δ‐10 Torr P_ET_CO_2_ conditions, respectively (BPM; *p* = 0.110). MAP was not significantly different between Δ 0, Δ‐5, and Δ‐10 Torr P_ET_CO_2_ conditions (mmHg; *p* = 0.726, see Table 1). MAP did not significantly change between each BL period and its corresponding VS (*p* > 0.05), confirming no concomitant effects on CBV from changes in MAP (and thus conductance; e.g., Battisti‐Charbonney et al., 2011). Because participants were coached to hyperventilate, their V̇_I_ increased between the Δ 0, Δ‐5, and Δ‐10 Torr P_ET_CO_2_ conditions from 12.9 ± 2.8 to 15.7 ± 3.3 and 20.6 ± 5.7, respectively (L/min; *p* < 0.001). Resting (Δ 0) P_ET_CO_2_ levels were 36.7 ± 3.2 Torr. In a randomized order, these levels were coached to Δ‐5 and Δ‐10 Torr P_ET_CO_2_ (*p* < 0.001). Δ‐5 Torr P_ET_CO_2_ had a mean BL of 31.6 ± 3.9 Torr and Δ‐10 Torr P_ET_CO_2_ had a mean BL of 26.0 ± 4.0 Torr. During normocapnia, we estimated that participants had a pHa of 7.43 ± 0.04, with each graded step of hypocapnia increasing estimated pHa, with Δ‐5 Torr P_ET_CO_2_ having an estimated pHa of 7.46 ± 0.06 and Δ‐10 Torr P_ET_CO_2_ having an estimated pHa of 7.58 ± 0.08. There were no significant differences (<±2 Torr) in P_ET_CO_2_ between BL, VS or across the entire VS protocol (*p* > 0.05), confirming that P_ET_CO_2_ was relatively well‐maintained at target values throughout the assessment of NVC. Last, because participants were coached to hyperventilate, their P_ET_O_2_ increased between the Δ 0, Δ‐5, and Δ‐10 Torr P_ET_CO_2_ conditions from 84.2 ± 7.0 to 87.9 ± 7.3 and 102.9 ± 5.4, respectively (Torr; *p* < 0.001).

### 3.2 NVC analysis

#### 3.2.1 Assessment of PCAv

**FIGURE 2:**
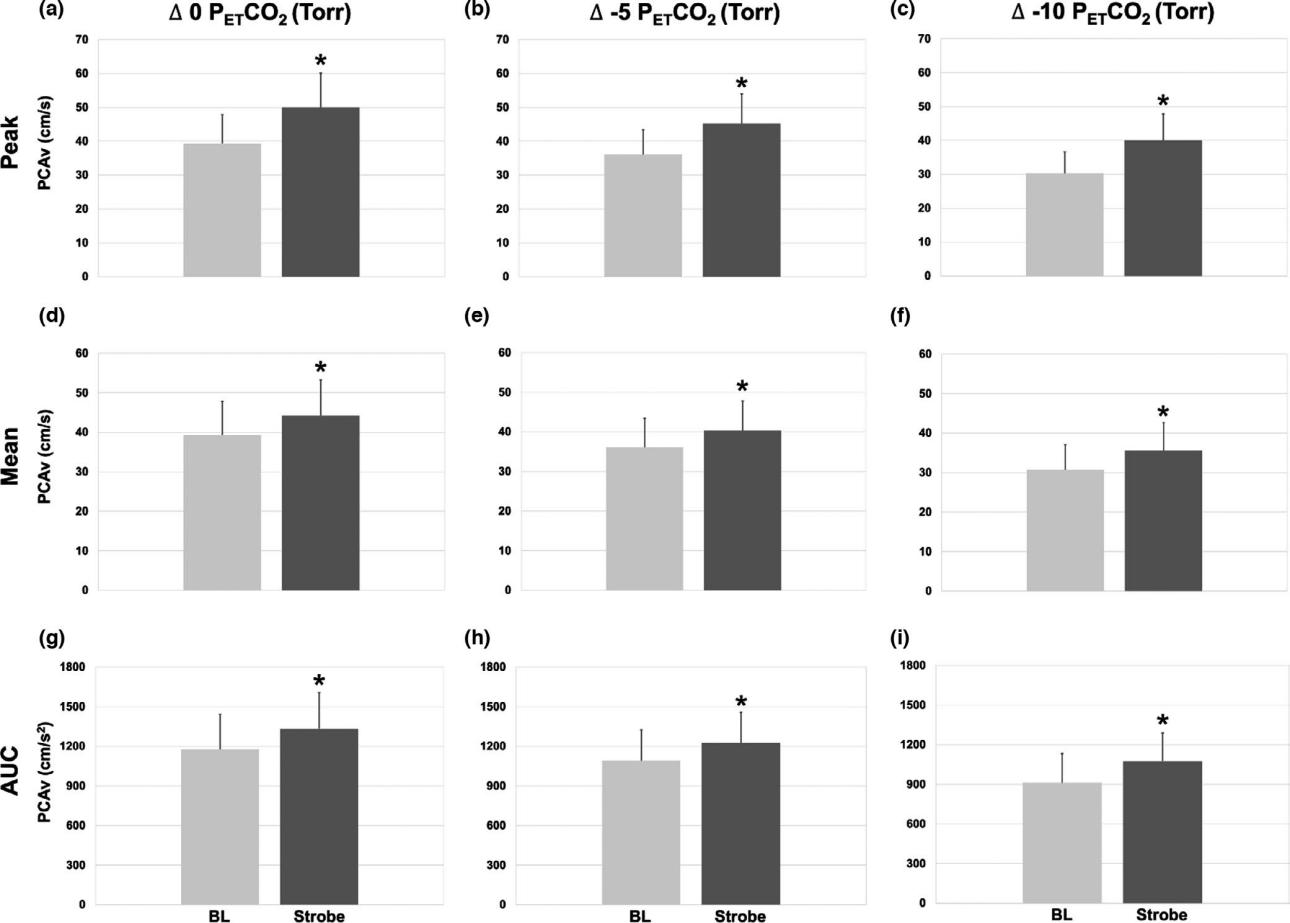
PCAv at baseline (BL) and during visual stimuli (strobe) in all three conditions (Δ0, Δ‐5, Δ‐10). (a–c) Absolute PCAv at BL compared with peak PCAv during visual stimulation across each condition. (d–f) Absolute mean PCAv at BL compared with mean PCAv during visual stimulation across each condition. (g–i) Absolute PCAv tAUC during BL compared with PCAv tAUC during visual stimulation. PCAv peak represents visually identified peak within each of the five strobe light stimulation. PCAv mean was collected from a total average of strobe light sections. Values are presented as mean ± SD. (*) indicates values significantly different from baseline (*p* < 0.001). These data confirm the presence of an NVC response under each condition, for each NVC analysis metric

A near‐even split of left and right PCAs were used, with nine left PCAs and eight right PCAs. Mean values of BL PCAv during Δ 0 P_ET_CO_2_, Δ‐5 P_ET_CO_2_, and Δ‐10 P_ET_CO_2_ were 39.3 ± 8.6, 36.2 ± 7.3, and 30.7 ± 6.4 (cm/s; *p* = 0.001), respectively. A reduction in BL suggests the vasoconstriction resulting from relative hypocapnia, as expected. Despite reductions in BL PCAv, an NVC response was still preserved. Figure 1b illustrates a sample tracing of one participant's derived mean PCAv over the course of one intervention and the corresponding NVC response to VS. A significant NVC response was elicited during normo‐ and hypocapnic conditions across each NVC metric (see Figure 2).

#### 3.2.2 NVC responses

Figure 2a–c illustrate each of the 1‐min BLs and the corresponding intervention's peak PCAv (*p* < 0.001). Figure 2a illustrates a PCAv change from 39.3 ± 8.6 cm/s to 50.0 ± 10.2 cm/s (Δ 10.7 ± 3.4 cm/s) during Δ 0 P_ET_CO_2_. Figure 2b illustrates a PCAv change from 36.2 ± 7.3 cm/s to 45.3 ± 8.7 cm/s (Δ 9.1 ± 3.5 cm/s) during Δ‐5 P_ET_CO_2_. Figure 2c illustrates a PCAv change from 30.7 ± 6.4 cm/s to 40.00 ± 7.9 cm/s (Δ 9.3 ± 2.6 cm/s) during Δ‐10 P_ET_CO_2_.

Figure 2d–f illustrates a 1‐min BL of each intervention to its corresponding mean PCAv (*p* < 0.01). Figure 2d illustrates a PCAv from 39.3 ± 8.6 to 44.3 ± 9.0 cm/s (Δ 5.0 ± 2.2 cm/s) during Δ 0 P_ET_CO_2_. Figure 2e illustrates a mean PCAv from 36.2 ± 7.3 to 40.3 ± 7.5 cm/s (Δ 4.2 ± 2.7 cm/s) during Δ‐5 P_ET_CO_2_. Figure 2f illustrates a mean PCAv from 30.7 ± 6.4 to 35.6 ± 7.0 cm/s (Δ 3.7 ± 4.91 cm/s) during Δ‐10 P_ET_CO_2_.

Similar to the mean Δ PCAv, there were increases in AUC Δ PCAv during each VS (*p* < 0.001; Figure 2g–i). Figure 2g illustrates the AUC PCAv from 1177.7 ± 266.4 to 1333.2 ± 273.8 cm/s^2^ (Δ 155.6 ± 81.6 cm/s) during Δ 0 P_ET_CO_2_. Figure 2h illustrates the AUC PCAv from 1092.7 ± 233.0 to 1226.3 ± 230.4 cm/s (Δ 126.6 ± 74.0 cm/s) during Δ‐5 P_ET_CO_2_. Figure 2i illustrates the AUC PCAv from 914.4 ± 218.6 to 1077.4 ± 212.8 cm/s (Δ 151.7 ± 74.9 cm/s) during Δ‐10 P_ET_CO_2_.

#### 3.2.3 Comparison of NVC responses in hypocapnia

**FIGURE 3:**
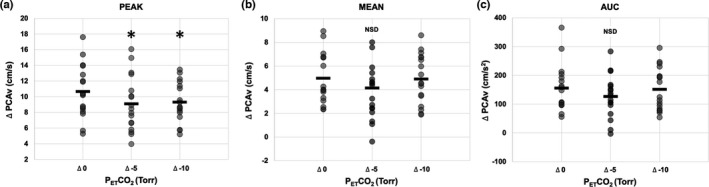
A comparison of NVC response magnitude for each metric during normal P_ET_CO_2_ (Δ0) and both P_ET_CO_2_ conditions (Δ‐5, Δ‐10). (a) The magnitude (Δ) of the peak change in PCAv from baseline during visual stimulation compared across all three conditions. (b) The magnitude (Δ) of the mean change in PCAv from baseline during visual stimulation compared across all three (c). The magnitude (Δ) of the tAUC in PCAv from baseline during visual stimulation compared across all three conditions. (*) indicates values significantly different from baseline (*p* < 0.05)

A comparison of response magnitude for each NVC metric is illustrated in Figure 3. A small but significant decrease in Δ peak PCAv was observed between Δ 0 and the Δ‐5 and Δ‐10 Torr P_ET_CO_2_ conditions (*p* = 0.044; see Figure 3a). However, no further differences were observed for Δ peak PCAv response magnitude between Δ‐5 and Δ‐10 Torr P_ET_CO_2_ interventions (*p* = 0.736). In addition, time‐to‐peak between the three CO_2_ trials (normocapnia, ‐5 and ‐10 Torr) was 14.5 ± 3.6, 13.4 ± 2.9, and 12.7 ± 4.2 s, respectively (*p* = 0.3; data not shown). No significant differences were observed Δ mean PCAv or Δ tAUC PCAv between any of the conditions (*p* = 0.193 and *p* = 0.19, respectively; see Figure 3b–c, respectively.

## 4 DISCUSSION

We assessed the magnitude of NVC responses to a standardized VS against the background of acute, incremental hypocapnia in 17 healthy participants. Our study revealed a small, but significant decrease in peak NVC response at both Δ‐5 Torr P_ET_CO_2_ and Δ‐10 Torr P_ET_CO_2_, while there were no significant reductions in either mean or tAUC NVC responses between normo‐ and hypocapnic conditions. The results of this study demonstrate that NVC is remarkably stable during acute respiratory alkalosis. In addition, this study illustrates the complexity of interpreting the NVC response and highlights the need to perform standardized quantification metrics to assess NVC (e.g., peak, average, and tAUC) to assess the comprehensive temporal and special NVC response.

### 4.1 The effects of hypocapnia on NVC magnitude

This study is one of the few that has investigated the isolated effects of acute hypocapnia and respiratory alkalosis on the NVC response, and the only study that introduced the interventions of incremental, acute hypocapnic steps without the presence of confounding variables in a laboratory setting. These results confirm findings from Szabo et al. (2011), who controlled participants’ ventilation instead of P_ET_CO_2_, showing that hypocapnia blunts the peak NVC response at ~Δ‐10 Torr P_ET_CO_2_. The results of the present study used a more specific control of P_ET_CO_2_, ensuring greater control of the hypocapnic stimulus, revealing a significant decrease in peak PCAv response during Δ‐5 Torr P_ET_CO_2_ with no further reduction at Δ‐10 Torr P_ET_CO_2_, compared to normocapnia. However, we did not observe a similar trend in the mean PCAv or tAUC response magnitudes (Figure 3b,c), which showed no significant difference between hyperventilation interventions.

Aside from demonstrating the relative stability of NVC in acute respiratory alkalosis, our data suggest that it is important to utilize standardized metrics in assessing NVC magnitude, such that meaningful comparisons can be made between studies. Similar to the present study, Leacy et al. (2018) measured peak, mean, and tAUC NVC response magnitudes during incremental ascent to 4240 m. In contrast, Szabo et al. (2011) exclusively reported on peak PCAv response during VS. The NVC response is complex due to the diversity of mediators, pathways, and redundant mechanisms which regulate vessel tone and the hemodynamic response, such as neurons, astrocytes, and endothelial cells (Attwell et al., 2010). Incorporating a standardized procedure for data analysis when investigating NVC might allow greater comparability between studies (Squair et al., 2020).

### 4.2 Potential mechanisms

The largest contributor to the NVC response appears to be neuronal NO, which when inhibited pharmacologically, decreased neurovascular reactivity by an average of 64% across 11 in vivo animal studies (Hosford & Gourine, 2019). A more recent study found that pharmacological NOS inhibition in humans decreased the peak NVC response ~30%, while having no effect on the mean NVC response, further illustrating multiple mechanisms underlying NVC (Hoiland et al., 2020). In addition, Fathi et al. (2011) found that during in vitro assessment of human cerebral microvascular endothelial cells, hypocapnia significantly reduced NO production from endothelial cells, while astrocyte‐mediated NO production was not affected. However, this later study was performed in an isohydric hypocapnic cell culture with a controlled pH (7.36–7.4), thus offering little insight into the effects of acute hypocapnia in vivo, where an acid–base disturbance is evident.

Despite BL cerebral blood flow being lower with graded hypocapnia (see Table 1 and Figure 2), NVC from each new BL value was mostly unchanged with hypocapnia, aside from a mild but significant reduction in the transient peak (but not time‐to‐peak), potentially affected by hypocapnia‐mediated acute respiratory alkalosis. The results reported here and by Szabo et al. (2011), who measured capillary blood pH, implicate acute alkalosis as an underlying mechanism for the blunted peak NVC magnitude, however mild. Without similar attenuation for the time‐to‐peak, mean, and AUC NVC magnitude, the extended NVC response beyond the transient peak is likely controlled through differential mechanisms not impacted by acute changes in PCO_2_ or pHa. Specifically, the initial rise in PCAv during VS is thought to be neural feedforward NO signaling pathways, whereas feedback metabolic signaling is thought to contribute to the longer‐term, sustained elevation in PCAv (Hoiland et al., 2020; Iadecola et al., 2017; Phillips et al., 2016). Thus, the acute hypocapnia and associated respiratory alkalosis only mildly affected the initial feed‐forward NVC response, whereas the sustained feedback from metabolism‐dependent signaling appeared unaffected, given the overall NVC response was stable between CO_2_ levels.

Studies at HA, where individuals are chronically hypocapnic, show no difference in NVC response (Caldwell et al., 2017; Leacy et al., 2018). During acute hypocapnia individuals are alkalotic, whereas during chronic hypocapnia, like that experienced with prolonged sojourns at altitude, the kidneys compensate for the respiratory alkalosis with a relative metabolic acidosis, returning blood pH back toward normal values (Barker et al., 1957; Leacy et al., 2018; Zouboules et al., 2018). Additionally, the hypoxia and hypocapnia experienced at HA may have antagonistic physiological effects, thus mitigating the blunted NVC response to hypocapnia due to the vasodilatory effects of hypoxia. Previous studies have also investigated the effect of hypercapnia on NVC, but found no changes in the NVC response (Rosengarten et al., 2003). These findings may suggest that the effects of CO_2_ and acid–base disturbance on NVC may be direction‐dependent. In addition, the relatively small decrease in peak ΔPCAv observed in the present study suggests that acute changes in PCO_2_ and pHa play only a small role in influencing the NVC response from a BL and appear to affect the transient peak response only.

### 4.3 Methodological considerations

In the present study, estimations of PaCO_2_ were made from P_ET_CO_2_, which is less accurate than arterial blood sampling, although PaCO_2_ is limited to point measurements with arterial blood draws. Previous studies found that P_ET_CO_2_ can reliably estimate PaCO_2_ in healthy individuals, without the need for specialized techniques or risk of infection and participant discomfort (Mcswain et al., 2010; Petersson & Glenny, 2014; Tymko et al., 2015). Specifically, measures of end‐tidal PCO_2_ (P_ET_CO_2_) during hyperventilation are reflected in PaCO_2_ (e.g., Krapf et al., 1991; Szabo et al., 2011). Thus, PaCO_2_ values can be estimated using non‐invasive P_ET_CO_2_ measurements (Tymko et al., 2015). During normocapnia, using mean bicarbonate values obtained in our lab, we estimated that participants had a pHa of ~7.43 ± 0.04, similar to normal physiological levels. Each graded step of hypocapnia increased pHa, with Δ‐5 Torr P_ET_CO_2_ having an estimated pHa of ~7.46 ± 0.06 and Δ‐10 Torr P_ET_CO_2_ having an estimated pHa of ~7.58 ± 0.08, suggesting appreciable acute respiratory alkalosis in our participants.

We employed coached voluntary hyperventilation to achieve our graded hypocapnic steps. Although this method was successful in eliciting our desired intervention, P_ET_O_2_ was increased slightly during hyperventilation. However, the increases in P_ET_O_2_ were small (~15 Torr), and within the physiological range (85–105 Torr; see Table 1). Indeed, increases in P_ET_O_2_ will theoretically cause vasoconstriction, in the same direction as the decreases in P_ET_CO_2_. However, other studies have suggested that although there may be some interaction between PO_2_ and PCO_2_ (Mardimae et al., 2012), the known reductions in CBF in the hyperoxic direction are small (e.g., Willie et al., 2012). Coached hyperventilation is a standard technique utilized in studies investigating the effects of hypocapnia on CBF (e.g., Willie et al., 2012), and there is no other way to reduce CO_2_ non‐invasively in humans. Thus, although these small increases in P_ET_O_2_ may have contributed to BL vasoconstriction in hypocapnia (see Table 1), the influence of these concomitant changes in blood gases on CBF are in the same direction, and likely negligible.

Percent change values were not used in the present study, opting instead to present raw data (absolute and deltas), given that as expected, BL PCAv was affected by prevailing P_ET_CO_2_ in each trial. Percent changes from lower BL variables in hypocapnia would likely over‐estimate observed changes, even if absolute ΔPCAv are unchanged. Thus, Szabo et al.'s study (2011), who only reported %‐changes in peak responses, may have reported exaggerated responses in relative hypocapnia compared to normocapnia. Using mean responses across the VS might be a better representation of ΔPCAv than peak, since it would account for the entire response during the VS. Szabo et al. (2011) only reported peak responses from the first 5‐s of the NVC response, while the present study used peaks from the entire 30‐s interval, wherever they occurred, similar to the previous literature (Leacy et al., 2018). However, the peak NVC response occurred early in the VS periods, and given that the peak response was reduced significantly, but not mean or tAUC PCAv response, suggests the possibility that acid–base disturbance may act only on specific mechanisms responsible for the immediate visually evoked hemodynamic response (e.g., Hoiland et al., 2020).

### 4.4 Potential significance

Developing a clear understanding of how blood gas perturbations and acid–base disturbances affect cerebrovascular control has obvious application to both high‐altitude acclimatization (e.g., Caldwell et al., 2017; Leacy et al., 2018) and during acute or chronic alterations in respiratory gases (e.g., Hoiland et al., 2016). In addition, NVC is impaired in several pathologies, including traumatic head injuries and stroke (Hinzman et al., 2014; Lin et al., 2011). Thus, elucidating the relationship between CBF regulation and hypocapnia and/or alkalosis could improve our fundamental understanding of CBF regulation in health and disease. In addition, the methods of eliciting and analyzing NVC responses vary between studies. Future studies should follow a standardized methodology to improve comparability between studies. In our study, alterations in peak, mean, and tAUC NVC in the PCA during VS resulted in varied deductions to the NVC response during hypocapnic conditions. Peak responses may be affected by acute alkalosis, while the sustained response observed with mean and tAUC analysis are not, suggesting differential and redundant mechanisms mediating NVC (e.g., Hoiland et al., 2020). The differences in outcome measures reported here depending upon analytical technique highlight the importance of a standardized model of studying NVC and quantification.

## 5 CONCLUSION

We aimed to isolate the effects of step‐wise acute hypocapnia and respiratory alkalosis on NVC magnitude. We observed a small but statistically significant reduction in NVC magnitude in peak PCAv, during both Δ‐5 Torr P_ET_CO_2_ and Δ‐10 Torr P_ET_CO_2_ interventions. However, no further reductions were observed between Δ‐5 Torr P_ET_CO_2_ and Δ‐10 Torr P_ET_CO_2_, suggesting a floor effect in acute respiratory alkalosis. In contrast, using mean and AUC PCAv metrics, there were no significant differences in NVC magnitude between BL and the two hypocapnic conditions. Our data illustrate that there may be differences in conclusions drawn depending on the analysis metric utilized. Thus, investigators are encouraged to utilize a more comprehensive and standardized analysis approach when assessing NVC in order to obtain a comprehensive overview of the NVC response.

## CONFLICT OF INTEREST

None to report.

## AUTHOR CONTRIBUTIONS

TJB, JKL, JRGK, AC, JRD, and ALM ‐ data collection and analysis; TAD, KDOH, NGJ, and RJAW ‐ study design and data interpretation; TAD ‐ funding, lab equipment support, student training; all co‐authors ‐ intellectual contributions and edited and approved the final manuscript.

## Data Availability

The data that support the findings of this study are available from the corresponding author upon reasonable request from a qualified researcher.
